# Water reuse and growth inhibition mechanisms for cultivation of microalga *Euglena gracilis*

**DOI:** 10.1186/s13068-021-01980-4

**Published:** 2021-06-05

**Authors:** Mingcan Wu, Ming Du, Guimei Wu, Feimiao Lu, Jing Li, Anping Lei, Hui Zhu, Zhangli Hu, Jiangxin Wang

**Affiliations:** 1 Shenzhen Key Laboratory of Marine Bioresource and Eco-Environmental Science, Shenzhen Engineering Laboratory for Marine Algal Biotechnology, Guangdong Provincial Key Laboratory for Plant Epigenetics, College of Life Sciences and Oceanography, Shenzhen University, Shenzhen, 518060 China; 2 Key Laboratory of Optoelectronic Devices and Systems of Ministry of Education and Guangdong Province, College of Optoelectronic Engineering, Shenzhen University, Shenzhen, 518060 China; 3 College of Food Engineering and Biotechnology, Hanshan Normal University, Chaozhou, 521041 China

**Keywords:** Microalgae, *Euglena gracilis*, Water reuse, Growth inhibitor, Humic acid, Metabolomics

## Abstract

**Background:**

Microalgae can contribute to more than 40% of global primary biomass production and are suitable candidates for various biotechnology applications such as food, feed products, drugs, fuels, and wastewater treatment. However, the primary limitation for large-scale algae production is the fact that algae requires large amounts of fresh water for cultivation. To address this issue, scientists around the world are working on ways to reuse the water to grow microalgae so that it can be grown in successive cycles without the need for fresh water.

**Results:**

In this study, we present the results when we cultivate microalgae with cultivation water that is purified and reused. Specifically, we purify the cultivation water using an ultrafiltration membrane (UFM) treatment and investigate how this treatment affects: the biomass and biochemical components of the microalgae; characteristics of microalgae growth inhibitors; the mechanism whereby potential growth inhibitors are secreted (followed using metabolomics analysis); the effect of activated carbon (AC) treatment and advanced oxidation processes (AOPs) on the removal of growth inhibitors of *Euglena gracilis*. Firstly, the results show that *E. gracilis* can be only cultivated through two growth cycles with water that has been filtered and reused, and the growth of *E. gracilis* is significantly inhibited when the water is used a third time. Secondly, as the number of reused water cycles increases, the Cl^−^ concentration gradually increases in the cultivation water. When the Cl^−^ concentration accumulates to a level of fivefold higher than that of the control, growth of *E. gracilis* is inhibited as the osmolality tolerance range is exceeded. Interestingly, the osmolality of the reused water can be reduced by replacing NH_4_Cl with urea as the source of nitrogen in the cultivation water. Thirdly, *E. gracilis* secretes humic acid (HA)—which is produced by the metabolic pathways for valine, leucine, and isoleucine biosynthesis and by linoleic acid metabolism—into the cultivation water. Because HA contains large fluorescent functional groups, specifically extended π(pi)-systems containing C=C and C=O groups and aromatic rings, we were able to observe a positive correlation between HA concentration and the rate of inhibition of *E. gracilis* growth using fluorescence spectroscopy. Moreover, photosynthetic efficiency is adversely interfered by HA, thereby reductions in the synthetic efficiency of paramylon and lipid in *E. gracilis*. In this way, we are able to confirm that HA is the main growth inhibitor of *E. gracilis*. Finally, we verify that all the HA is removed or converted into nutrients efficiently by AC or UV/H_2_O_2_/O_3_ treatments, respectively. As a result of these treatments, growth of *E. gracilis* is restored (AC treatment) and the amount of biomass is promoted (UV/H_2_O_2_/O_3_ treatment).

**Conclusions:**

These studies have important practical and theoretical significance for the cyclic cultivation of *E. gracilis* and for saving water resources. Our work may also provide a useful reference for other microalgae cultivation.

**Supplementary Information:**

The online version contains supplementary material available at 10.1186/s13068-021-01980-4.

## Background

Unicellular eukaryotic microalgae are a diverse and ubiquitous group of plants that are a promising biomass source and a biological feedstock [1, 2]. However, the use of microalgae as a feedstock is limited because a large amount of water is needed for its cultivation, and this negatively affects economic viability and environmental sustainability [3]. Wastewater treatment is currently being used to recycle the water that is necessary for microalgae cultivation [4]. It has been reported that to produce 1 kg of algae biomass, 1564 L of water are required under pond conditions [5]. Reusing cultivation water can reduce water usage and nutrient requirements as well as the need for algal wastewater treatment [3]. Consequently, water reuse after algae harvesting is essential for the economic viability of the microalgae industry and for environmental sustainability.

The effects of water reuse on algae growth are different across algae taxa [6]. The most researched taxa are green algae, diatoms, cyanobacteria, haptophytes, eustigmatophytes, chrysophytes, and xanthophytes [3]. However, among the genus of *Euglena* spp. algae, especially, there have been no reports of *E. gracilis* cultivation with reused water. *E. gracilis* is a unicellular flagellated alga characterized by the absence of a cell wall. It produces a wide variety of bioactive compounds such as paramylon, carotenoids, tocopherols, euglenophycin, and lipids. It has tremendous potential for metabolic engineering and commercialization [7]. Therefore, a deeper understanding of water reuse in the cultivation of the microalga *E. gracilis*, and the mechanisms underlying its growth-inhibiting secretions, are urgently needed.

After the microalgae assimilate nutrients ions, unabsorbed counter ions such as Cl^−^, Na^+^, and K^+^ from NH_4_Cl, NaHCO_3_, and KH_2_PO_4_, respectively, can accumulate in the cultivation water. When these ions accumulate, the osmotic pressure of the cultivation water increases, thereby inhibiting microalgae growth [8, 9]. Therefore, finding a suitable medium, which can balance the osmotic pressure between microalgae and cultivation water, is important for improving the effects of water reuse.

In addition to accumulated ions, excreted metabolites, such as dissolved organic matter (DOM) from microalgae is considered to be the main cause of negative biomass growth [3, 6, 10]. For *Scenedesmus* sp. LX1, DOM concentrations between 6.4 and 25.8 mg/L in reused water resulted in a decrease in the maximum algae cells density and the maximum growth rate by 50–80% and 35–70%, respectively [11]. The DOM in that study was classified into two fractions: hydrophobic or hydrophilic. Each of these fractions was further classified as acids, neutrals, or bases for a total of six fractions. In that study, all six fractions showed inhibited algal growth. Moreover, Lu et al. [10] who also used this fractionation approach, reported that the DOM of *Scenedesmus acuminatus* in the reused water included palmitic acid and octadecanoic acid, both of which inhibited the growth of this algae species. Although many studies have attempted to characterize the growth inhibitor present in DOM, it is unclear which major metabolic pathways within microalgae cells regulate and secrete these inhibitory substances into the cultivation water.

Recently, many researchers have tried to use traditional methods of wastewater treatment on microalgae cultivation water. Zhang et al. [12] reported that the removal of DOM with activated carbon (AC) in the reused water of cultivated *Nannochloropsis oceanica* moderately reduced growth inhibition and lipid accumulation. Moreover, the AC treatment significantly increased the final dry weight of *S. acuminatus* to 2.33 ± 0.04 g/L, which was almost the same as the dry weight obtained after growth in fresh media [13]. Advanced oxidation processes (AOPs) are another type of treatment technology in which organic pollutants are destroyed by powerful oxidizing agents [14, 15]. O_3_, UV/H_2_O_2_ have been successfully applied in the treatment reused water for the cultivation of *Scenedesmus* sp. LX1 [16] and *S. acuminatus* GT-2 [17], respectively. To date, few studies have investigated the removal of DOM by AC or AOPs in reused water of cultivated *E. gracilis*.

In this study, the main objectives were: (1) to identify the effect of treatment with ultrafiltration membrane (UFM) on the biomass and biochemical components of cultivated *E. gracilis*; (2) to identify the characteristics of the growth inhibitors in reused water and uncover the mechanism whereby potential inhibitors are secreted by *E. gracilis* via metabolomics analysis; (3) and to evaluate the effect of AC and AOPs on the removal of growth inhibitors.

## Results and discussion

### Effects of reused water on the growth of *E. gracilis*

**Fig. 1:**
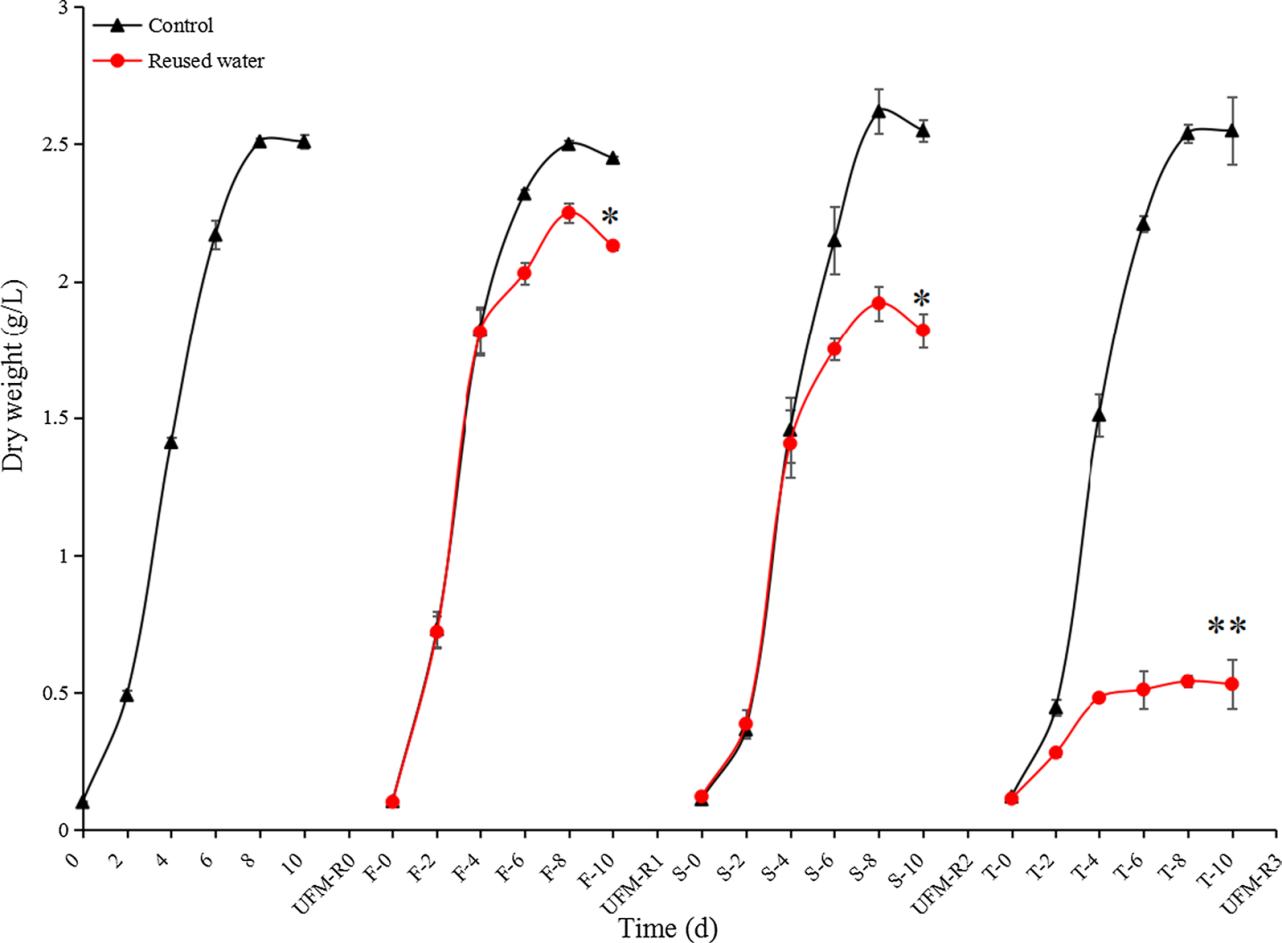
Effects of reused water on the growth of *E. gracilis*. UFM-R0, -R1, -R2, and -R3 represent the number of times water is reused and treated with UFM; The letters F, S, and T combined with 0, 2, 4, 6, 8, and 10 represent the number of days for algae cultivation under the first (F), second (S), and third (T) conditions of reused water, respectively. Asterisk represents *p* < 0.05; double asterisk represents *p* < 0.01. The values are represented by mean ± SD, where *n* = 3

Since the UFM can cut off substances with a molecular weight ≥ 50 kDa, viruses, bacteria, macromolecular proteins, polysaccharides, and other substances can be filtered out [10, 12], so only unconsumed ions and DOM remain in the reused water. This study found that the DW of algae cells gradually decreased in the reused water with successive cycles. The DW of algae with each cycle of cultivation decreased by 13.1% (UFM-R1, *p* < 0.05), 28.6% (UFM-R2, *p* < 0.05), and 79.2% (UFM-R3, *p* < 0.01) compared to the control group on the last day of cultivation (Fig. 1). By the third cycle of cultivation, the growth of algae cells had been severely inhibited. This suggests that the increase in the presence of growth inhibitors with successive cycles of water reuse reduces algal growth beyond a tolerable range. This phenomenon is similar to the growth inhibition observed for other microalgae such as *S. acuminatus* [10], *Chlorella*. SDEC-18 [18], and *N. oceanica* [12]. We postulate that accumulated ions and algae cells secretions of DOM in the reused water are the main factors that inhibit the growth of *E*. *gracilis*.

### The effect of accumulated ions on the growth of *E. gracilis*

**Fig. 2:**
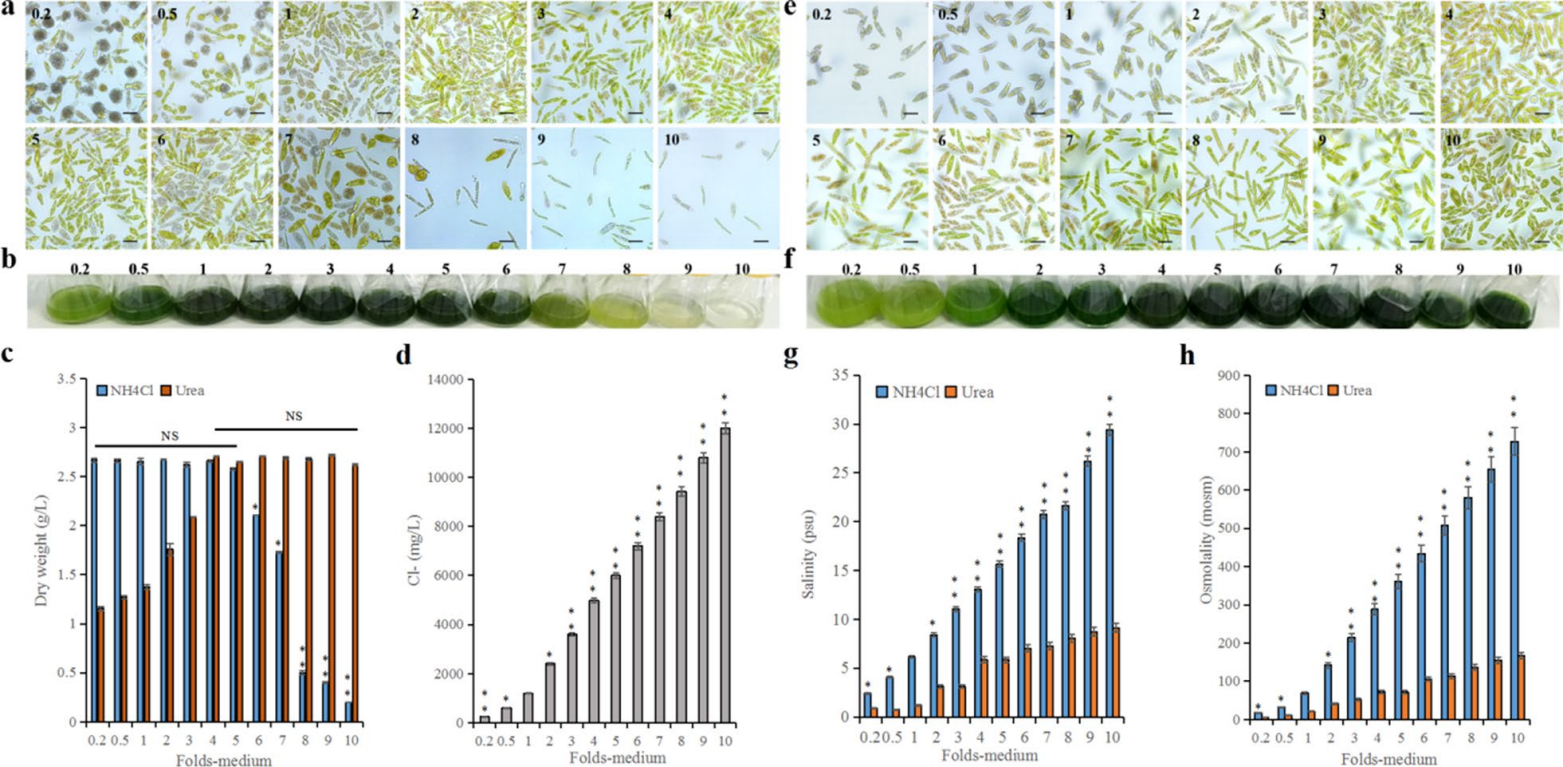
Effects of accumulated ions on the growth of *E. gracilis*. **a** morphological changes and relative algae cells density (NH_4_Cl as nitrogen source). **b** Cytochrome changes in algae cells (NH_4_Cl as nitrogen source). **c** The biomass of algae cells cultured under NH_4_Cl and urea as nitrogen sources. **d** The Cl^−^ ions concentration. **e** The morphological changes and relative algae cells density (urea as a nitrogen source). **f** Cytochrome changes of algae cells (urea as a nitrogen source). **g** The salinity and **h**, osmolality of the medium with NH_4_Cl and urea as nitrogen sources, respectively. “Folds-medium” on the *x*-axis in **c**, **d**, **g**, and **h** represent different multiples of PEM medium concentration. NS, asterisk, and double asterisk represent *p* > 0.05, *p* < 0.05, and *p* < 0.01, respectively. The values are represented by mean ± SD, where *n* = 3

Microalgae can selectively absorb some types of ions from inorganic nutrients and assimilate them into their own organic matter. But some ions such as Cl^−^ and Na^+^, cannot be absorbed by microalgae. These residual ions, which accumulate in reused water, can destroy the balance of osmolality of the microalgae, thereby inhibiting their growth [8, 9]. This study found that when algae cells were cultivated in more than eightfold the concentration of PEM medium (NH_4_Cl as the nitrogen source) compared to the control, the relative cell density was lower, algal cytochromes were almost absent, and the algae cells were elongated (Fig. 2a, b). When the concentration of the culture medium was within fivefold of the control medium, the DW of algal cells was about 2.7 g/L, which was not significantly different from the control group (*p* > 0.05). However, when the concentrations of the medium were increased to above fivefold that of the control medium, the DW gradually decreased. In fact, when the concentration of the medium was increased by eight-, nine-, and tenfold, the DW of cells decreased by 81.3% (*p* < 0.01), 85.0% (*p* < 0.01), and 92.5% (*p* < 0.01), respectively (Fig. 2c). In addition, the Cl^−^ concentration was increased as the medium concentration increases, the maximal concentration was reached 11,996.4 mg/L in the tenfold medium (Fig. 2d), suggesting that accumulated Cl^−^ in the medium may be a key growth inhibitor of *E. gracilis*.

To prove the above hypothesis, we used urea instead of NH_4_Cl as the nitrogen source (equal nitrogen content) and found that as the concentration of urea increased, the DW of algae cells increased significantly. When the concentration of the culture medium was four to tenfold, the biomass of algal cells was stably maintained at about 2.7 g/L, and there was no significant difference among them (*p* > 0.05) (Fig. 2c). In addition, when urea was used as a nitrogen source, the cells appeared to be fuller. With an increase in the concentration of the culture medium, the relative cells density increased and the relative content of chlorophyll gradually increased as well (Fig. 2e, f). At the same time, the salinity and osmolality in the medium were much lower than those of the medium using NH_4_Cl as the nitrogen source. For example, the salinities at tenfold medium concentration were 29.4 psu versus 9.1 psu for NH_4_Cl versus urea, respectively. The osmolalities under these conditions were 727.0 mosm versus 167.0 mosm for NH_4_Cl versus urea, respectively. The salinity value for NH_4_Cl was 3.2-fold greater than urea (Fig. 2g, *p* < 0.01), and the osmolality for NH_4_Cl was 4.3-fold greater than urea (Fig. 2h, *p* < 0.01). These results show that the growth of algae cells was not inhibited with a fivefold increase in salinity (< 15.6 psu) and osmolality (< 361.1 mosm) of the medium, and we can confirm that during the UFM-R3 culture cycle, the growth of *E. gracilis* was not hindered by the accumulated ions in the reused water. This phenomenon has also been confirmed by the cultivation of *S. acuminatus* in reused water [10, 19]. Our work also showed that the growth of *E. gracilis* had a certain tolerance range to ions. If this tolerance range was exceeded, the growth of *E. gracilis* was inhibited. In addition, it was determined that the traditional medium PEM with NH_4_Cl as the nitrogen source was not suitable for the continuous recycling of cultivation water or for batch-fed cultivation of *E. gracilis* (such as heterotrophic batch-fed fermentation). When urea is used, it serves as an ideal nitrogen source because it reduces the salinity and osmolality in the culture medium.

### Identification of growth inhibitors in *E. gracilis* secretions

**Fig. 3:**
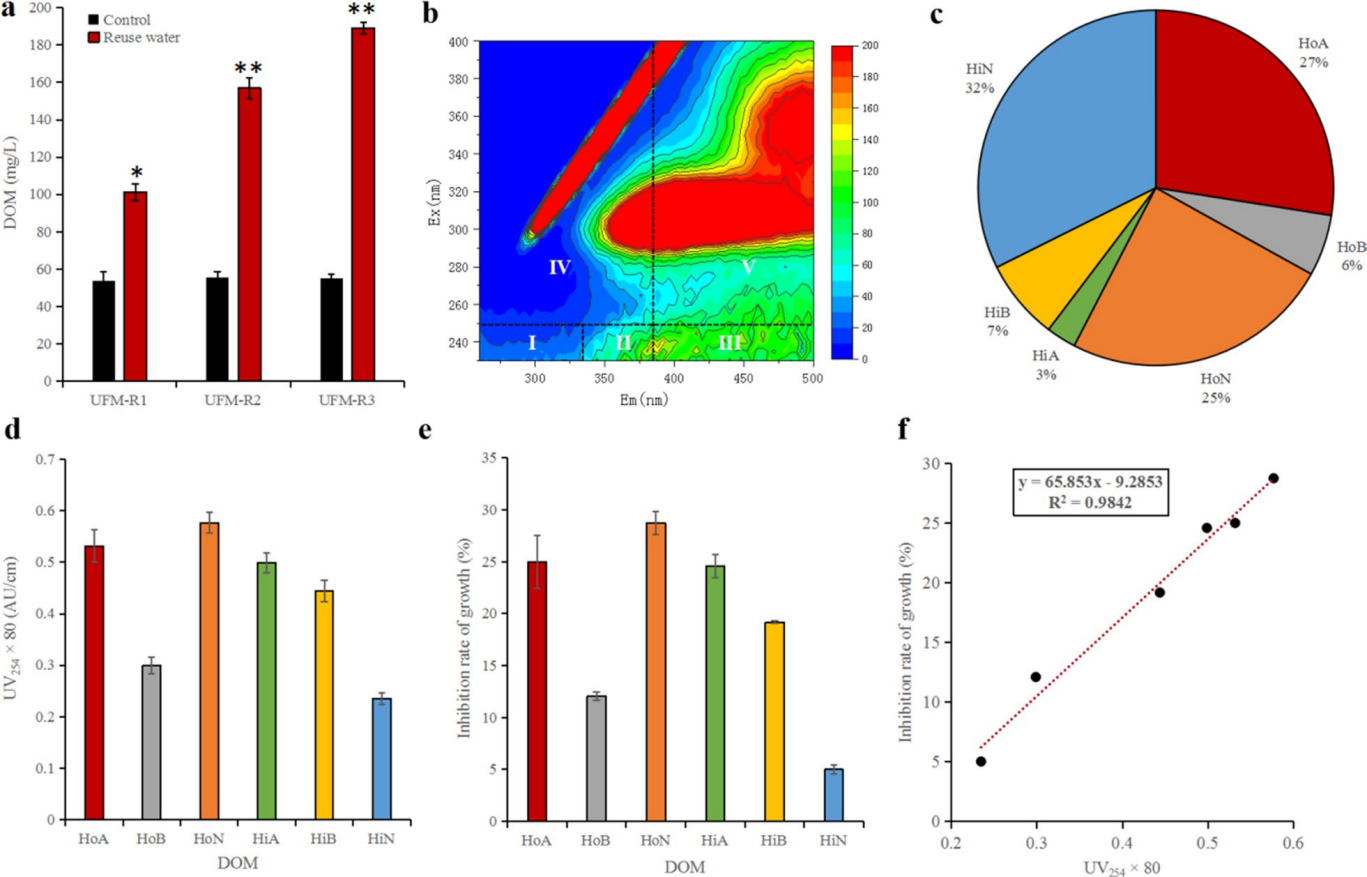
Identification of growth inhibitors in *E. gracilis* secretions. **a** The content of DOM in reused water by using ultrafiltration membrane treatment. **b** Three-dimensional (3D) fluorescence excitation–emission matrix (FEEM) spectra of DOM (I and II, AP, aromatic proteins; III, FA, fulvic acid-like, IV, SMBM, soluble microbial by product-like material; V, HA, humic acid). **c** Percent of 6 DOM fractions from reused water of *E. gracilis*. **d** UV_254_ of 6 DOM fractions in 80-fold medium (*HoB* hydrophobic bases, *HiN* hydrophilic neutrals, *HoA* hydrophobic acids, *HiB* hydrophilic bases, *HiA* hydrophilic acids, *HoN* hydrophobic neutrals). **e** The inhibition rate of growth (IG%) of *E. gracilis* with stress from different DOM fractions. **f** The relationship between UV_254_ and IG% (inset: trendline linear equation and *R*^2^ value). Asterisk represents *p* < 0.05, double asterisk represents *p* < 0.01. The values represent mean ± SD, where *n* = 3

The growth of microalgae is not affected by certain osmotic pressures for reused water, so growth inhibitors may exist in the DOM secreted by microalgae. However, some DOM can promote the growth of microalgae while some have an inhibitory effect on microalgae growth [3], so further study of these DOM characteristics is required. This study also found that *E. gracilis* continuously secreted DOM during the culture process. By the time UFM-R3 was reached, the DOM concentration was 189.21 mg/L, while the control group contained only 54.92 mg/L DOM, a 3.4-fold difference (*p* < 0.01) (Fig. 3a). This indicates that, at elevated concentrations, DOM may have an inhibitory effect on the growth of *E. gracilis*.

3D-FEEM fluorescence spectroscopy is fast and has excellent selectivity and sensitivity for fluorescent substances [20]. Therefore, in this study, this technique was used to identify the types of DOM secreted by algae cells. Chen et al. [21] used 3D-FEEM fluorescence spectroscopy to identify the following substances in the DOM present in cultivation water: aromatic proteins (AP), fulvic acid-like substances (FA), soluble microbial by product-like material (SMBM), and HA. We used those assignments to determine which types of DOM were present in our cultivation water samples (they are labeled with roman numerals in the spectrum in Fig. 3b. See caption b). It can be seen from Fig. 3b that the abundance of organic compounds with fluorescent signals in the reused water from high to low as: HA, SMBM, FA, and AP. Our spectra showed that HA was the potential main type of DOM present in *E. gracilis* secretions.

In order to further identify the growth inhibitors, we divided the DOM into six major fractions using fractional distillation (Fig. 3c). The percentages from high to low were: HiN (32%), HoA (27%), HoN (25%), HiB (7%), HoB (6%), and HiA (3%). From this result, we know that the DOM is mainly composed of HiN, HoA, and HoN, suggesting that HA, a potential inhibitor of *E. gracilis*, is composed of these organic acids. We also know that the slope of peaks in a UV spectrum (at 254 nm, given in AU/cm) for organic matter represents the content of organic functional groups that contribute to fluorescence, such as C=C bonds, C=O bonds, and aromatic rings. The importance of ultraviolet spectra for detecting pollutants in the water treatment process was described by Altmann et al. [22]. In this study, we tested the UV_254_ of 80-fold-concentrated DOM and found that the fluorescence intensity from high to low was: HoN (0.58 AU/cm), HoA (0.53 AU/cm), HiA (0.50 AU/cm), HiB (0.44 AU/cm), HoB (0.30 AU/cm), and HiN (0.24 AU/cm) (Fig. 3d). The inhibition of growth, IG%, of *E. gracilis* for each of these organic substances were: 28.8%, 25.0%, 24.6%, 19.2%, 12.1%, and 5.0% (Fig. 3e), respectively. It suggests that all of these DOM fractions can inhibit the growth of *E. gracilis*, especially HoN, HoA, and HiA. In addition, it is obvious that the UV_254_ absorption value is linearly related to the IG% for *E. gracilis* based on the graph in Fig. 3f for which *R*^2^ = 0.9. The degree of inhibition was positively correlated with the content of luminescent functional groups in the DOM. Based on these results, we confirmed that all fractions of DOM with C=O bonds, C=C bonds, and aromatic rings have an inhibitory effect on the growth of *E. gracilis*. In other words, inhibiting the growth of *E. gracilis* mainly depended on the concentration of different fractions.

According to the above results, DOM mainly includes HA, which was mainly composed of three organic compounds: HiN, HoA, and HoN (Fig. 3b). However, when using U254 signal to characterize these organics, in addition to HoA and HoN with relatively high signal intensity, HiA, HiB, and HoB also had an inhibitory effect on the growth of *E. gracilis*, suggesting that inhibitors other than HA may also be present in the recycled culture media. These growth inhibitory factors may be derived from SMBM, FA, and AP (Fig. 3b). These hydrophilic/hydrophobic fractions also have inhibitory effects on the growth of microalgae, such as FA has been proven to have an inhibitory effect on *Scenedesmus* species [13], indicating that this fraction, as well as HA with its highly fluorescent signals, may be potential inhibitors. However, both the concentration and the UV_254_ signal intensity of HiA, HiB, and HoB were lower than HoA and HoN derived from HA. In addition, although the concentration of HiN derived from HA was relatively higher, it obviously reduced the inhibitory effect on the growth of *E. gracilis*. Therefore, this study finally confirmed that the main growth inhibition of *E. gracilis* was HA, and the hydrophobic HoA and HoN organics fractions with higher content and higher UV_254_ signal intensity played a key inhibitory role. Lu et al. [10] only fractionated HoN-containing fatty acids and showed that they have an inhibitory effect on the growth of *S. acuminatus*. In addition, Zhang et al. [11] showed that all of the fractions could inhibit the growth of *Scenedesmus* sp. LX1, especially, HiB, HoB, and HiA. However, HoN and HoA showed the strongest inhibition of *E. gracilis*. This suggests that different microalgae may have different tolerances to different classifications of DOM. This scientific problem requires further research.

### The influence of *E. gracilis* secretions on its physiology and biochemistry

**Fig. 4:**
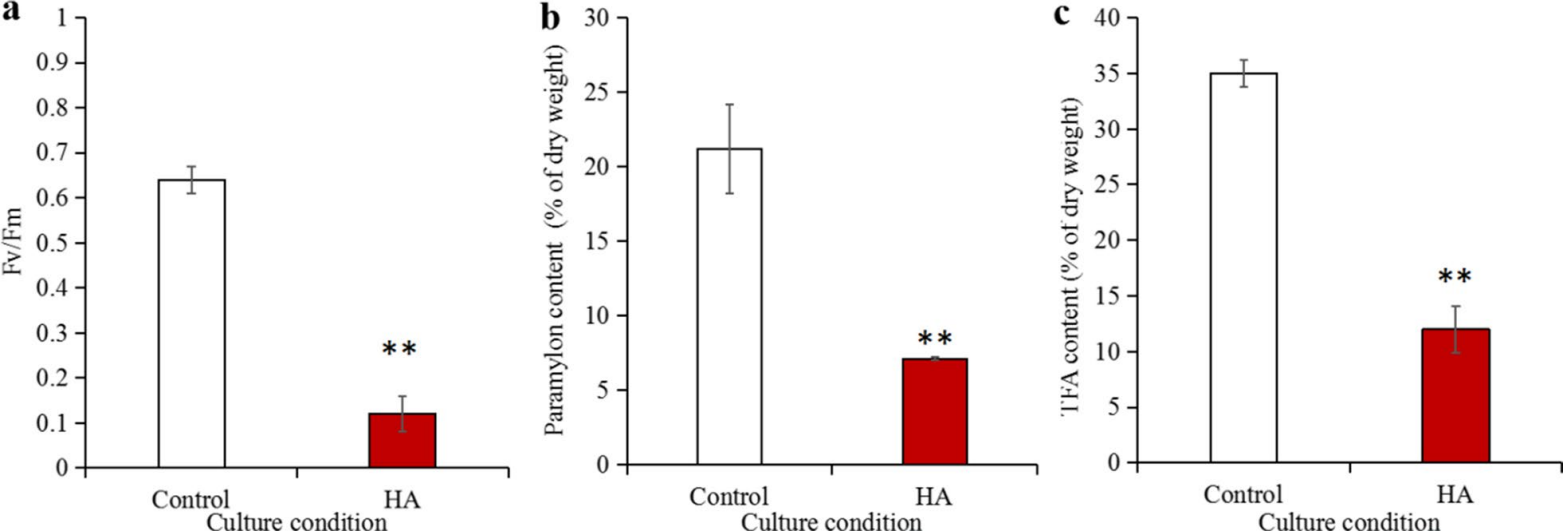
The effect of self-secreted DOM on (**a**) Fv/Fm ratio, (**b**) paramylon content, and (**c**) TFA content of *E. gracilis*. *TFA* total fatty acid, *HA* humic acid. Double asterisk represents *p* < 0.01. The values represent mean ± SD, where *n* = 3

The Fv/Fm ratio reflects the ability of microalgae to dissipate, absorb, and transmit light energy during photosynthesis. It is a useful parameter that indicates physiological state and growth rate, and is also an internal probe of the relationship between microalgae and their environment [13, 23, 24]. The Fv/Fm ratio for algae cells was only 0.12 in water containing HA, while that of the control group was 0.64, which is 5.3-fold difference (Fig. 4a, *p* < 0.01). From this result, it is obvious that HA significantly reduces the algae cell’s photosynthetic efficiency. Similarly, studies on *S. acuminatus* [13] and *Arthrospira platensis* [23] also showed comparable Fv/Fm reductions when cultivated in reused water, which means that HA has a negative effect on the photosynthetic system of these microalgae too. Thus, this impact has a certain universality.

The paramylon content and TFA content of *E. gracilis* in the experimental group containing DOM were 7.1% and 12.2%, respectively, while the control group was 21.2% and 35.2%. Both of these values were significantly lower than the control group, which showed a decrease of 66.5% (Fig. 4b, *p* < 0.01), and 65.3% (Fig. 4c, *p* < 0.01), respectively. These results were confirmed for the TFA of *Scenedesmus* sp. LX1 [11]. These results indicate that the HA secreted by *E. gracilis* may interfere with its own photosynthesis, and that this leads to inhibition of the synthesis of organic matter in the algae cells. The mechanism behind this process is worthy of our in-depth study in the future.

### Study on the mechanism of *E. gracilis* growth inhibition by its own secretions

When UHPLC–QTOF-MS was used to detect metabolites in *E. gracilis* cells and cultivation media, the range of metabolites detected in negative ion mode was greater than that in positive ion mode, so this study only analyzes metabolites that were observed in negative ion mode to describe the mechanism whereby algae cells secrete DOM. With this analysis, we observed 4130 metabolites (Additional file 2). These metabolites were analyzed by PCA and OPLS-DA, and we can see clear separation between intracellular (IEG) and extracellular (EEG) metabolites (Additional file 1: Fig. S2, S3), indicating that there were significant differences in the metabolites in these two groups. When the OPLS-DA permutation test was performed on the data, the categorical variable Y was randomly changed 1000 times (Additional file 1: Fig. S4) and the original model *R*^2^*Y* was equal to 1, indicating that the established model conforms to the real situation for the sample data. The original model had a *Q*^2^ value equal to 0.997, which is very close to 1. This means that if a new sample were added to the model, it would fall within the existing distribution of data points. In general, the original model is robust and can explain the difference between the two sets of samples well. No overfitting was required to fit our data to it.

**Fig. 5:**
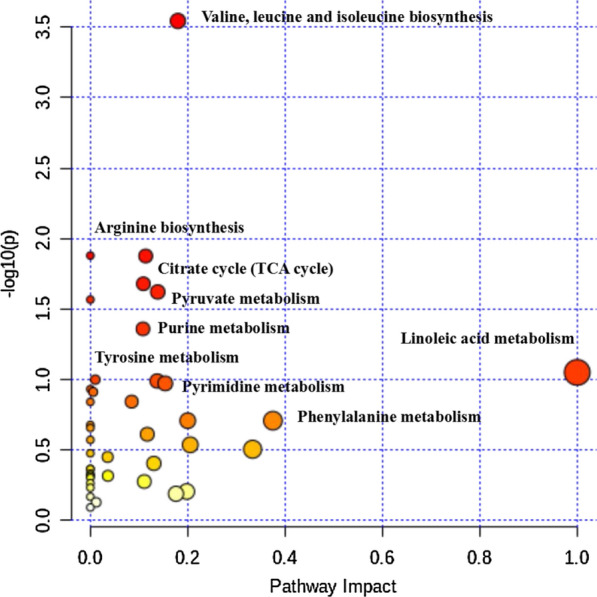
The top nine KEGG pathways of groups EEG and IEG are presented in the bubble chart. Each bubble in the bubble chart represents a metabolic pathway. The *X*-axis of the bubble and the bubble scale indicate the influence factor of the pathway in the topology analysis. The larger the size, the greater the influence factor; the *Y*-axis and the color of the bubble indicate the enrichment analysis. *P* value (take the negative natural logarithm, namely − log10(*p*), the redder the color, the smaller the *P* value, the more significant the enrichment degree. IEG, EEG represent intracellular and extracellular metabolites, respectively

**Fig. 6:**
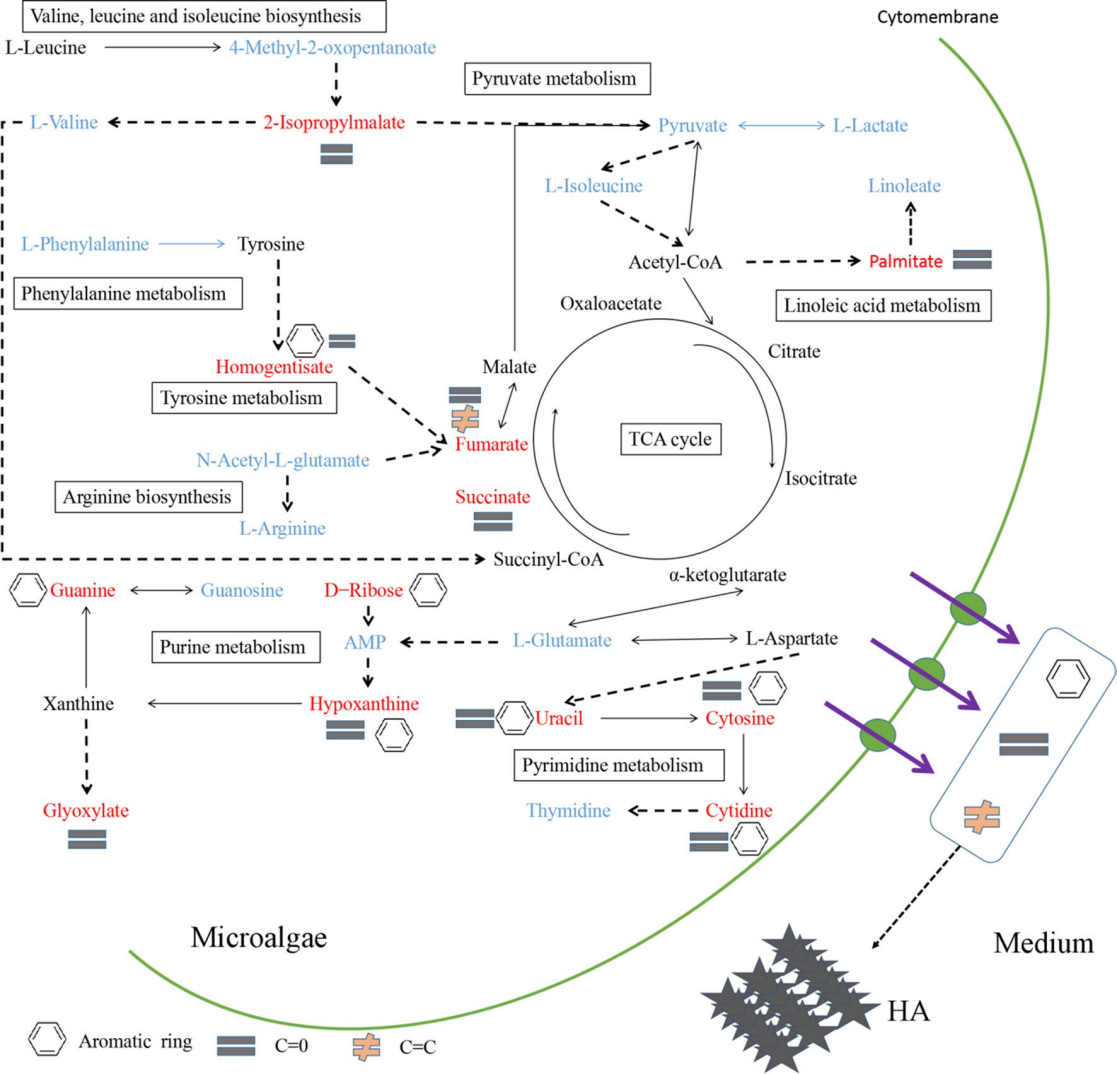
The schematic diagram of the mechanism of *E. gracilis* secretion of growth inhibitors. Red font indicates that the relative concentration of metabolites outside the cell is greater than that inside the cell, and blue font indicates that the relative concentration of metabolites outside the cell was less than that inside the cell. The solid line represents the direct chemical reaction, and the dashed line represents the indirect chemical reaction. HA represents humic acid

This study used VIP > 1 and a *P*-value < 0.05 to screen metabolites, and 108 different metabolites were obtained (see Additional file 2). According to the heat map cluster analysis, the relative concentration of 69 and 39 metabolites in the EEG and IEG were up-regulated, respectively (Additional file 1: Fig. S5). After these metabolites were annotated by the KEGG database, important metabolic pathways were screened according to their position and role in the relevant metabolic pathways (Additional file 2). According to the bubble chart, there are nine main metabolic pathways that are relevant: valine, leucine, and isoleucine biosynthesis; linoleic acid metabolism; arginine biosynthesis; the TCA cycle; pyruvate metabolism; purine metabolism; tyrosine metabolism; pyrimidine metabolism; and phenylalanine metabolism (Fig. 5). Among these, the first two are the key metabolic pathways. Some of the metabolites in these metabolic pathways were highly expressed inside the cell, and some were highly expressed outside the cell, and the latter group of metabolites may be secreted from the cell into the cultivation water. Three pathways—linoleic acid metabolism, the TCA cycle, and valine, leucine, and isoleucine biosynthesis—involve C=O and C=C bonds, while purine and pyrimidine metabolism contribute aromatic rings and C=O bonds. These metabolites accumulate in the medium and gradually become HA, which contains various functional groups (Fig. 6).

Palmitic acid was one of the metabolites secreted by *S. acuminatus* to inhibit growth [10]. Similarly, this study found that the palmitic acid in linoleic acid metabolism was higher in concentration in the culture medium. Therefore, it was further proved that palmitic acid was also one of the key factors that inhibit the growth of *E. gracilis*. 2-Isopropylmalate is an intermediate product of valine, leucine, and isoleucine biosynthesis. Excessive secretion of this intermediate product from algal cells into the cultivation medium may also inhibit the growth of *E. gracilis*. However, exactly how this intermediate metabolite inhibits the growth of *E. gracilis* is a question that requires in-depth research in the future.

HA containing multiple functional groups can complex iron ions that are essential for photosynthesis in microalgae. However, Sun et al. [25] found that the underlying mechanism of the inhibitory effect for cyanobacteria was not to reduce the bioavailability of iron, but to inhibit the oxidative damage of cells mediated by peroxidase-mediated. More and more evidence shows that HA could directly interact with certain large plants and algae through their different functional groups, thereby interfering with photosynthesis and growth. Due to their low molecular weight (< 50 kDa), these substances can easily pass through cell membranes. When these quinone-containing metabolites enter the chloroplast, they interfere with the electron transport processes of photosynthesis [26, 27]. In fact, the toxic effects of quinones on the growth and photosynthesis of *Scenedesmus* strains have been confirmed [28]. In addition, we have previously found that there was no significant difference between the experimental group and the control group under heterotrophic conditions containing HA (data not disclosed) and the Fv/Fm ratio was significantly reduced (Fig. 4a), which means that these inhibitors may primarily attack the photosynthetic system of the *E. gracilis* chloroplast. However, no metabolites related to quinones were found in the different metabolites screened in this study (Additional file 1: Fig. S5), indicating that the photosynthetic machinery of *E. gracilis* was not affected by quinones. Moreover, it is possible that different functional groups (e.g. C=C and C=O bonds, aromatic rings) interfere with the electron transport processes of photosynthesis. How these functional groups in the compounds secreted by different metabolic pathways interfere with the photosynthetic system of microalgae requires further in-depth study.

### Removal of growth inhibitors

**Fig. 7:**
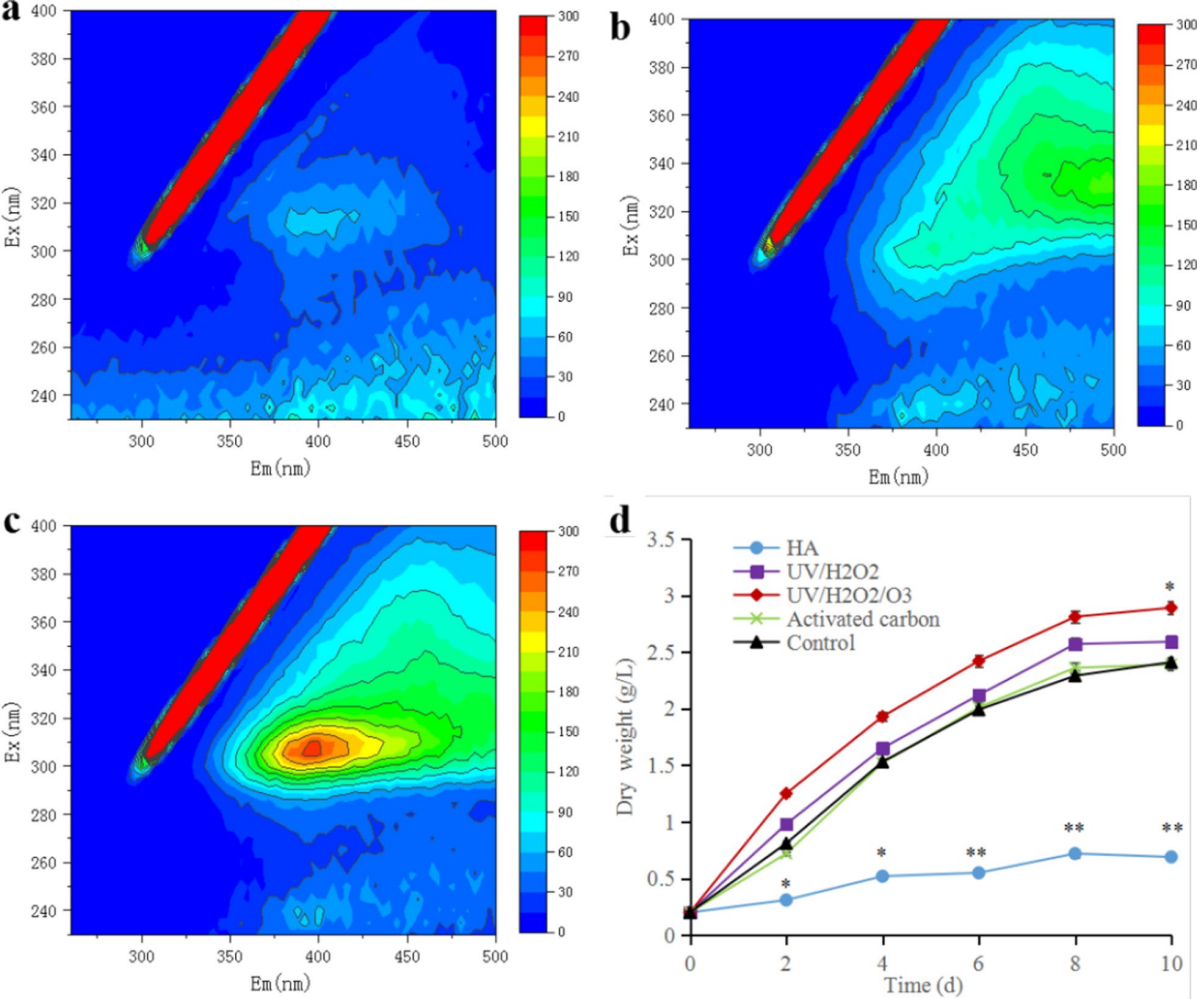
Removal of growth inhibitors. 3D-FEEM spectra of HA after: (**a**) activated carbon; (**b**) UV_254_/H_2_O_2_/O_3_-treatment. (**c**) UV_254_/H_2_O_2_-treatment; (**d**) Dry weight (DW) of *E. gracilis* after different treatments. The regions in these spectra are assigned to various substances found in DOM in Fig. 3b and its caption. HA represents humic acid. Asterisk represents *p* < 0.05. The values represent mean ± SD, where *n* = 3

Markiewicz et al. [29] have confirmed that DOM in sewage is adsorbed effectively by AC. The fluorescence spectrum after AC treatment showed that the fluorescence signal was very weak (Fig. 7a), indicating that almost all of the HA that can fluoresce had been removed. In addition, the growth curve for the experimental group was almost the same as that of the control group (Fig. 7d). By the time of the last day of culture, the DWs of algal cells were 2.4 g/L (experimental group) and 2.4 g/L (control group), with no significant difference (*p* > 0.05). This indicates that AC is effective at completely adsorbing and removing substances that inhibit the growth of *E. gracilis.* Although the reused water of cultivated *N. oceanica* [12] *and S. acuminatus* [13] showed a relatively significant effect from AC treatment, the biomass obtained was slightly lower than the control group, indicating that some growth inhibitors could not be removed. However, AC effectively adsorbs growth inhibitors secreted by *E. gracilis* in this study. We would like to develop recyclable AC technology, such as biological AC, to increase the utilization rate so that it can be more convenient for large-scale reuse of water resources to cultivate *E. gracilis*.

AOPs have been widely used in the field of wastewater treatment. Oxidizers create a large number of free radicals under ultraviolet catalysis, such as hydroxyl radicals. These free radicals have strong oxidizing properties and can oxidize organic acids with unsaturated bonds [15]. According to the 3D-FEEM spectra of the reused water after AOP treatment, the fluorescence signal of the UV/H_2_O_2_/O_3_ group was weaker (Fig. 7b), followed by UV/H_2_O_2_ (Fig. 7c), indicating that the oxidation efficiency was higher with the participation of O_3_. In addition, the biomasses of the UV/H_2_O_2_/O_3_ group and the UV/H_2_O_2_ group on the last day were 2.89 g/L and 2.59 g/L, respectively, with the UV/H_2_O_2_/O_3_ experimental group significantly higher than the control group (*p* < 0.05). These results indicate that the advanced oxidation method not only eliminates the growth inhibitors, but may also oxidize these inhibitors into small organic molecules that could be absorbed by algae cells, thereby increasing their biomass. Our results show that the growth inhibitors were mainly HAs with luminescent functional groups (C=O and C=C bonds, aromatic rings). O_3_ and UV/H_2_O_2_ have been shown to work well for the treatment of *Scenedesmus* sp. LX1 [16] and *S. acuminatus* GT-2 [17] reused water, respectively. This study combines these two methods and shows that both methods together are more effective at removing growth inhibitors than either O_3_ or UV/H_2_O_2_ alone. Therefore, we believe that UV/H_2_O_2_/O_3_ is an ideal and efficient method for the removal of inhibitors of *E. gracilis*.

According to our previous research, the free radicals in the reused water after treatment with AOPs could also inhibit the growth of microalgae. Therefore, we need to optimize the AOP treatment process in the future by optimizing the treatment time, the concentration of the oxidizing agent, and the development of indicators for online detection of the concentration of free radicals in reused water (for example, the vitamin C reducing agent neutralization method). Use of AOPs is more conducive to the wide application of water reuse for algae cultivation. In addition, through these treatments, again it is clear that HA secreted by *E. gracilis* is a main growth inhibitor.

**Fig. 8:**
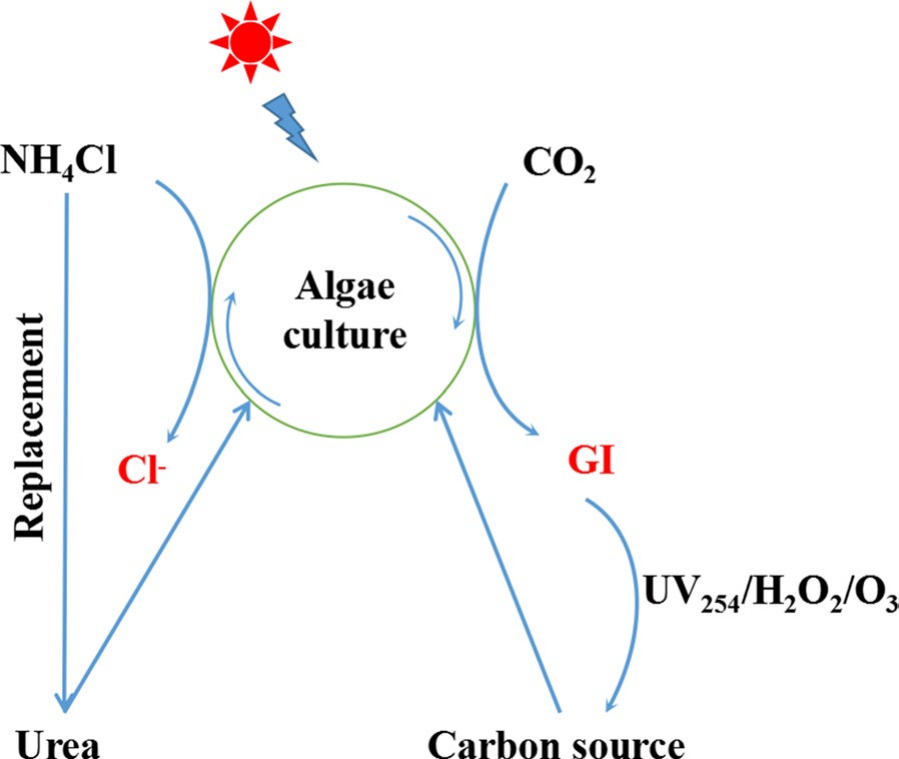
The cyclic culture model of *E. gracilis* under reused water conditions*.* GI represents the growth inhibitors secreted by microalga *E. gracilis*

Based on the above results, we have proposed a cyclic culture model for *E. gracilis* (Fig. 8). The conceptual model is optimal when urea replaces NH_4_Cl as a nitrogen source and the reused water is filtered through an UFM and then treated with UV_254_/H_2_O_2_/O_3_. This model improves the availability of reused water, reduces the cost of cultivation, and increases the biomass of microalga *E. gracilis*.

## Conclusion

Our study demonstrated that cultivation water used three times had a significant inhibitory effect on the growth of *E. gracilis*. We replaced NH_4_Cl with urea and observed a reduction in the osmotic pressure caused by Cl^−^ accumulation in the reused water, indicating that urea is an ideal nitrogen source. In addition, HA was identified as a main growth inhibitor of *E. gracilis*, and its content was positively related to the rate of growth inhibition. Moreover, we found that HA interfered with the photosynthetic efficiency of the algae and reduced the efficiency of paramylon and lipid synthesis. We determined that the key metabolic pathways for secreting these HA were valine, leucine, and isoleucine biosynthesis, and linoleic acid metabolism via metabolomics analysis. All HA been efficiently removed or converted into nutrients by AC or UV/H_2_O_2_/O_3_ treatment, respectively. As a result, the biomass has been recovered to the same levels as the control group (AC treatment) and even enhanced *E. gracilis* growth (UV/H_2_O_2_/O_3_ treatment). This result provides further confirmation that HA was a main growth inhibitor. An effective model for the cyclic culture of *E. gracilis* was thus proposed. These studies have important practical and theoretical significance for cyclic cultivation of *E. gracilis* or even other species of microalgae and for saving precious water resources.

## Materials and methods

### Microalgae strain and growth conditions

*Euglena gracilis* CCAP 1224/5Z was obtained from the Culture Collection of Algae and Protozoa (CCAP) and maintained in our lab at Shenzhen University [30]. This strain was cultured in a modified photoautotrophic *Euglena* medium (PEM) according to Cramer and Myers [31]. PEM includes 1.8 g/L NH_4_Cl, 0.6 g/L KH_2_PO_4_, 1.2 g/L MgSO_4_·7H_2_O, 0.02 g/L CaCl_2_·2H_2_O, 0.55 μg/L Na_2_EDTA·2H_2_O, 2.0 μg/L Fe_2_(SO_4_)_3_, 0.05 μg/L CuSO_4_·5H_2_O, 0.4 μg/L ZnSO_4_·7H_2_O, 1.3 μg/L Co(NO_3_)_2_·6H_2_O, 1.8 μg/L MnCl_2_·4H_2_O, 0.01 μg/L Vitamin B_1_, and 0.0005 μg/L Vitamin B_12_. The pH value of the PEM medium was 3.6 adjusted with 3 mol/L NaOH and 1 mol/L HCl. The microalgae cells were grown in 2-L glass column photobioreactors with a 10-cm internal column diameter. The photobioreactors contained 1.5 L PEM medium that was stirred with 0.2 μm-filtered mixed gas (2% CO_2_, v/v, gas flow rate = 6 L/min) and illuminated with an LED lamp at a light intensity of 150 μmol photons m^−2^ s^−1^ (24: 0 h light–dark cycle). The temperature of the cultivation was maintained at 25.0 °C.

### Water reuse

When the algae cells were cultured on day 10, the cultivation water sample (UFM-R0) was treated with ultrafiltration membrane (UFM) harvesting equipment [32] that was built in our laboratory (Additional file 1: Fig. S1). This equipment cuts off the molecular weight ≥ 50 kDa. One equivalent volume of the PEM nutrient medium was added to the UFM-R0 water sample. After sterilization, the solution was inoculated with microalgae to a final OD_750_ concentration of 0.2 for the microalgae suspension. After the first cultivation, water samples were cycled through this cultivation and UFM cycle three times and these subsequent cycles were named UFM-R1, R2, R3. Samples of the microalgae were taken every other day to monitor the cells’ dry weight (DW).

DW was measured according to the method described by Wu et al. [33]. Briefly, 5 mL microalgae suspension was filtered through a preheated (105 °C, 24 h), pre-weighed glass microfiber filter (Whatman GF/C, 47 mm, UK). The filters were washed twice, each with 50 mL of 0.5 mol/L NH_4_HCO_3_. The filters were weighed after drying at 105 °C for 24 h to reach a constant mass. DW was calculated using Eq. (1):

1 $$\mathrm{DW}\frac{\mathrm{g}}{\mathrm{L}}= \frac{{w}_{\mathrm{a}}-{w}_{\mathrm{b}}}{v},$$

where “*w*_a_” and “*w*_b_” are the mass of the filters at the end and start of cultivation, respectively, and “*v*” is the volume of the microalgae suspension. Finally, the reused water samples and the algae cells were kept at − 80 °C to prepare for the next study.

### The effect of accumulated ions on the growth of *E. gracilis*

Algae cells were cultured in PEM with nutrient concentrations of 10, 9, 8, 7, 6, 5, 4, 3, 2, 1, 0.5, and 0.2-fold, with 1.8 g/L NH_4_Cl or 1.0 g/L urea (both of them contained equal nitrogen content) as the nitrogen source. When the algae cells were cultured on day 10, the morphology of the algae cells was observed with an inverted microscope (Leica DMI8, Leica Microsystems, Germany). In addition, the DW, Cl^−^ concentration, salinity, osmolality, and the pigment changes of the algae cells in the culture medium were measured. Cl^−^ concentration was measured using a capillary ion chromatograph (ICS 5000 +, Dionex, Sunnyvale, CA, USA) [10]. The salinity of the culture medium was measured using an Orion STAR A329 multiparameter meter (G10919, Thermo Fisher Scientific, USA). Osmolality was determined by measuring the freezing point of the solution with an automatic osmometer (Osmomat 030, Gonotec, Germany) according to Hadj-Romdhane et al. [9]. The osmolality was calculated using the freezing point depression, Δ*T*(°C), according to the following Eq. (2):

2 $$\mathrm{Osmolality} \mathrm{mosm}=\frac{\Delta T(^\circ{\rm C} )}{1.858},$$

where 1.858 is the cryoscopic constant, which is equal to the freezing point depression of a solution with an osmolality of 1 osmol.

### The identification of *E. gracilis* growth inhibitors and quantitative analysis of their effect on growth

The differences in dissolved organic matter (DOM) content between the control (UFM-R0) and the reused water samples (UFM-R1, R2, R3) were measured using a total organic carbon analyzer (Multi N/C 2100, Analytik Jena, Germany).

DOM was determined with three-dimensional fluorescence excitation–emission matrix (3D-FEEM) spectrophotometry. Briefly, 3D-FEEM spectra were obtained using a fluorescence spectrophotometer (F-4500, Hitachi, Japan). The excitation (Ex) and emission (Em) slits were set to a bandpass of 5 nm. Ex wavelengths were scanned from 200 to 450 nm, and Em wavelengths were scanned from 220 to 550 nm. All of the 3D-FEEM spectral data were analyzed with Origin Pro 2018 software (https://www.originlab.com/origin).

The method of pretreated water sample was based on Leenheer [34]. The fractional method of DOM from water sample was optimized by Imai et al. [35] and Zhang et al. [36]. Briefly, the water sample was repeatedly passed through Amberlite XAD-8 resin at a flow rate of 5 mL/min 3 times, and then 2 bed volumes (BV) of 0.1 mol/L HCl were added to reverse the elution 3 times to obtain the hydrophobic bases (HoB). The pH of the water sample was adjusted to 2.0 using 0.1 mol/L HCl and 0.1 mol/L NaOH, then the water sample was passed through 3 columns with XAD-8 resin, D001(a microporous strong acidic ion exchange resin), and D201 (a macroporous strong base ion exchange resin) orderly. This sequence was repeated 3 times. After the water sample was passed through the resin, the remaining water sample contained only the hydrophilic neutrals (HiN). The XAD-8, D001, and D201 resins were back-eluted with 0.1 mol/L NaOH to obtain hydrophobic acids (HoA), hydrophilic bases (HiB), and hydrophilic acids (HiA), respectively. After the XAD-8 resin was air dried, ethanol was added and soxhlet extraction was run for 12 h to obtain the hydrophobic neutrals (HoN). All solvents were removed using rotary-evaporation (RV3, IKA, Germany). The volume of all DOM fractions was adjusted to 50 mL and transferred to centrifuge tubes. After diluting each group of DOM to a certain concentration, the percentage of the DOM in each fraction was measured by using a total N/C analyser (Multi N/C 2100, Analytik Jena, Germany). An absorbance value of 254 nm (UV_254_) of DOM in each fraction was measured by using a UV–Vis spectroscopic measurements (UV2350, UNICO, China).

The fractionated organic acids were added to fresh medium according to the percentage of DOM. After culturing the algae cells with this medium for 10 days, the DWs of the control and the experimental groups were measured. The inhibition rate of growth (IG%) was calculated using the following Eq. (3):

3 $$\mathrm{IG\%}=\frac{c-i}{c}\times 100\mathrm{\%},$$

where “*c*” is the DW of the control group and “*i*” is the DW of algae cells under the different fractionated DOM stressed conditions. Finally, the correlation analysis of UV_254_ and IG% were performed by using Origin Pro 2018 software.

### The effect of growth inhibitors on the Fv/Fm ratio, paramylon and total fatty acid content

After the fractionated DOM was diluted according to the DOM content in the UFM-R3 reused water, as described above, fresh medium was added, and then the algae cells were cultivated under the afore mentioned culture conditions. The Fv/Fm ratio, the paramylon and the total fatty acid (TFA) content of the algae cells were measured after the 10th day of cultivation.

The Fv/Fm ratio was determined by dividing the variable fluorescence (Fv) by the maximum fluorescence (Fm), according to the method of Sha et al. [13]. The algae cells were placed in a quartz cube and maintained in the dark for 3 min prior to measurement of Fv/Fm. The Fv/Fm ratio for the algae cells was then measured at room temperature using a PHYTO-ED fluorimeter (Walz, Effeltrich, Germany).

Paramylon content was quantified using the method of Takenaka et al. [37] and Wu et al. [30] with the following modification: 2 mL, 30 mmol/L of EDTA chelating agent was added to 15 mL the algae cells suspensions. After each cell suspension was centrifuged and freeze-dried, 10 mg freeze-dried algae powder and 5 mL of acetone were transferred to a 15 mL of centrifuge tube and shaken for 30 s, then placed in a shaker for 2 h. After the tube was centrifuged at 2000×*g* for 5 min, the supernatant was removed. 1.5 mL of 1% sodium dodecyl sulfate (SDS) solution was added to the tube, then the contents were transferred into a 1.5-mL centrifuge tube and heated in a water bath at 85 °C for 2 h. Again, the supernatant was removed after the centrifuge tube was centrifuged at 2000×*g* for 5 min. The precipitate was washed and centrifuged in 1 mL deionized water, then oven-dried at 70 °C to a constant mass. The resulting precipitate was paramylon. The paramylon content was calculated as shown in Eq. (4):

4 $$\mathrm{Paramylon content \%}=\frac{P}{\mathrm{DW}}\times 100\mathrm{\%}$$

where “*P*” and “DW” are the DWs of the paramylon and the algae powder, respectively.

TFA content was determined using the method of Wu et al. [38]. Briefly, about 10 mg lyophilized cell pellets were disrupted by grinding three times under liquid nitrogen in the presence of methanol, chloroform, and formic acid (20:10:1, v:v:v) to extract the lipids from the algal biomass. The quantity of TFA content in extracts was measured by using an Agilent 7890B gas chromatograph coupled with a 5977A mass spectrometer (GC–MS).

### The metabolic pathways of growth inhibitors secreted by *E. gracilis* were determined using metabolomics analysis

*E. gracilis* cells and cultivation water from UFM-R0 were collected and a metabolomics analysis was performed. The metabolites in the sample were extracted and analyzed according to the method mentioned by Wu et al. [38]. All of the metabolites were detected using ultra-high performance liquid chromatography coupled with quadrupole time-of-flight mass spectrometry (UHPLC–QTOF-MS). In this study, some metabolite peaks were detected after relative standard deviation noise reduction. Next, the missing values were increased by half of the minimum value. An internal standard normalization method was also employed in this data analysis. The final dataset containing the peak number, sample name, and normalized peak area was imported to a SIMCA16.0.2 software package (Sartorius Stedim Data Analytics AB, Umea, Sweden) for multivariate analysis. Data was scaled and logarithmically transformed to minimize the impact of both noise and high variance of the variables. After these transformations, principal component analysis (PCA), an analysis that reduces the dimensionality of the data, was carried out to visualize the distribution and the grouping of the samples. A 95% confidence interval in the PCA score plot was used as the threshold to identify potential outliers in the dataset. In order to visualize group separation and find significantly changed metabolites, orthogonal-projections-to-latent–structures discriminate analysis (OPLS-DA) was applied. Then a sevenfold cross-validation was performed to calculate the values of *R*^2^ and *Q*^2^. *R*^2^ indicates how well the data variance fits the model, and *Q*^2^ indicates how well a variable can be predicted. To check the robustness and predictive ability of the OPLS-DA model, 1000 permutations were further conducted. Afterward, the *R*^2^ and *Q*^2^ intercept values were obtained. The intercept value of *Q*^2^ represents the robustness and reliability of the model and the risk of overfitting (for the latter, smaller values are better). Furthermore, the value of variable importance in the projection (VIP) of the first principal component in OPLS-DA analysis was obtained. It summarizes the contribution of each variable to the model. The metabolites with VIP > 1 and *p* < 0.05 (Student’s *t*-test) were considered to be significantly changed. In addition, commercial databases including KEGG database (http://www.genome.jp/kegg/) and MetaboAnalyst (http://www.metaboanalyst.ca/) were used for metabolic pathway enrichment analysis. From these analyses, bubble diagrams and metabolic pathways were made.

### Removal of growth inhibitors

The methods of advanced oxidation that we used (UV/H_2_O_2_/O_3_ and UV/H_2_O_2_) were similar to the result of Hu et al. [16] and Wang et al. [17]. Briefly, an ozone generator, which produced O_3_ with a flow rate of 3000 mg/h, was fed into a solution containing 189.2 mg/L DOM (found in reused water UFM-R3) and 1% H_2_O_2_ for 2 h under UV_254_ ultraviolet lamp irradiation. This is the UV/H_2_O_2_/O_3_ experimental group. In the other set of experiments, O_3_ was not used, but all other conditions were the same. This is the UV/H_2_O_2_ experimental group. After all the treated water samples were freeze-dried, fresh medium was added to each. The method of AC filtration was similar to that of Sha et al. [13]. Briefly, the water sample containing the same UFM-R3 DOM concentration was filtered repeatedly through a chromatography column containing saturated AC (K04, Hainan Xingguang Active Carbon Co., LTD. China) for 2 h. Then 0.45-micron membrane filters were used to recover the water sample. DOM was characterized by using 3D-FEEM spectroscopy. Finally, the microalgae cells were cultured under the experimental conditions, and the DW of the algae cells was measured every other day.

### Statistical analysis

All DWs, Cl^−^ concentration, salinity, osmolality, DOM, UV_254_, IG%, Fv/Fm ratio, paramylon content, and TFA content tests were performed in triplicate and the average and standard deviations was reported. All data were statistically analyzed by Student’s *t*-test analysis to investigate the difference between the control and experimental groups. *p*-values of less than 0.01 (*p* < 0.01) were considered significantly different, *p* < 0.05 values were considered statistically different, and *p* > 0.05 values were considered not statistically different (NS) compared to the control groups.

## Supplementary Information

**Additional file 1**: **Fig. S1.** Microalgae harvesting equipment. a, microalgae suspension; b, ultrafiltration membrane; c, pre-concentrated microalgae; d, reused water. **Fig. S2. **PCA analysis for groups EEG and IEG. IEG, EEG represent intracellular and extracellular metabolites, respectively. **Fig. S3. **OPLS-DA analysis for group EEG and IEG. IEG, EEG represent intracellular and extracellular metabolites, respectively. **Fig. S4. **Permutation test for group EEG and IEG. IEG, EEG represent intracellular and extracellular metabolites, respectively. **Fig. S5. **Heatmap of hierarchical clustering analysis for group EEG and IEG. IEG, EEG represent intracellular and extracellular metabolites, respectively; * represents the metabolite can be annotated in KEGG database.

**Additional file 2**. The data of statistical analysis results, different metabolites, and pathway analysis from the group EEG and IEG.

## Data Availability

All data generated or analyzed in the present study are included in this article and in additional information.

## Abbreviations

UFM: Ultrafiltration membrane
AC: Activated carbon
AOPs: Advanced oxidation processes
HA: Humic acid
DOM: Dissolved organic matter
AP: Aromatic proteins
FA: Fulvic-acid-like substances
SMBM: Soluble microbial by product-like material
IEG: Intracellular
EEG: Extracellular
CCAP: Culture Collection of Algae and Protozoa
PEM: Photoautotrophic *Euglena* medium
3D-FEEM: Three-dimensional fluorescence excitation-emission matrix
Em: Excitation (Ex) and emission
DW: Cells’ dry weight
HoB: Hydrophobic bases
BV: Bed volumes
HoB: Hydrophobic bases
HiN: Hydrophilic neutrals
HoA: Hydrophobic acids
HiB: Hydrophilic bases
HiA: Hydrophilic acids
HoN: Hydrophobic neutrals
IG%: The inhibition rate of growth
TFA: The total fatty acid
Fm: The maximum fluorescence
GC–MS: Gas chromatograph coupled with a 5977A mass spectrometer
UHPLC–QTOF-MS: Quadrupole time-of-flight mass spectrometry
PCA: Principal component analysis
OPLS-DA: Orthogonal-projections-to-latent–structures discriminate analysis
VIP: The value of variable importance in the projection
NS: Statistically different
