# Supplementary material for: Water reuse and growth inhibition mechanisms for cultivation of microalga Euglena gracilis
Source: Biotechnol Biofuels. 2021 Jun 5;14:132. doi: 10.1186/s13068-021-01980-4 (PMC8180174; doi:10.1186/s13068-021-01980-4)
Supplement: Supplementary file 1 — Additional file 1: Fig. S1. Microalgae harvesting equipment. a, microalgae suspension; b, ultrafiltration membrane; c, pre-concentrated microalgae; d, reused water. Fig. S2. PCA analysis for groups EEG and IEG. IEG, EEG represent intracellular and extracellular metabolites, respectively. Fig. S3. OPLS-DA analysis for group EEG and IEG. IEG, EEG represent intracellular and extracellular metabolites, respectively. Fig. S4. Permutation test for group EEG and IEG. IEG, EEG represent intracellular and extracellular metabolites, respectively. Fig. S5. Heatmap of hierarchical clustering analysis for group EEG and IEG. IEG, EEG represent intracellular and extracellular metabolites, respectively; * represents the metabolite can be annotated in KEGG database. [file 13068_2021_1980_MOESM1_ESM.doc]

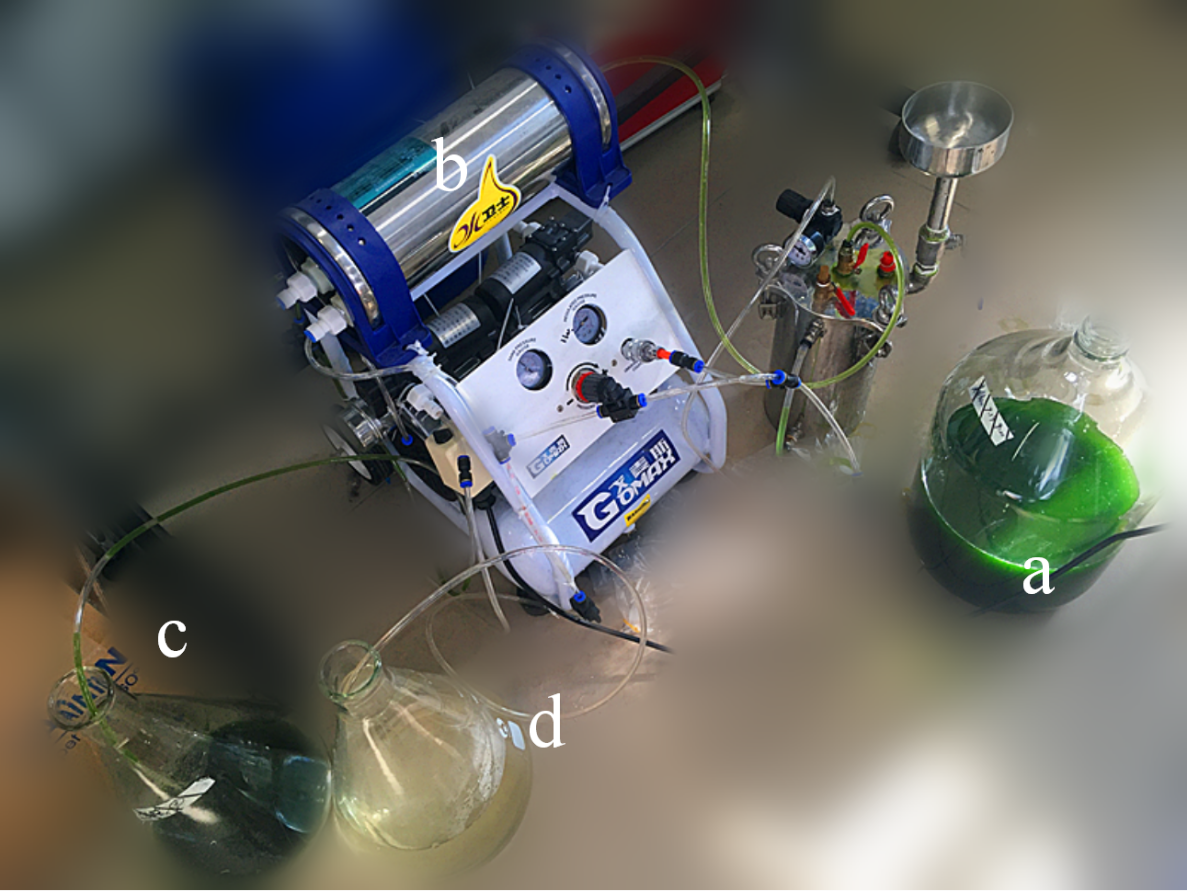


**Fig. S1.** Microalgae harvesting equipment. a, microalgae suspension; b, ultrafiltration membrane; c, pre-concentrated microalgae; d, reused water.


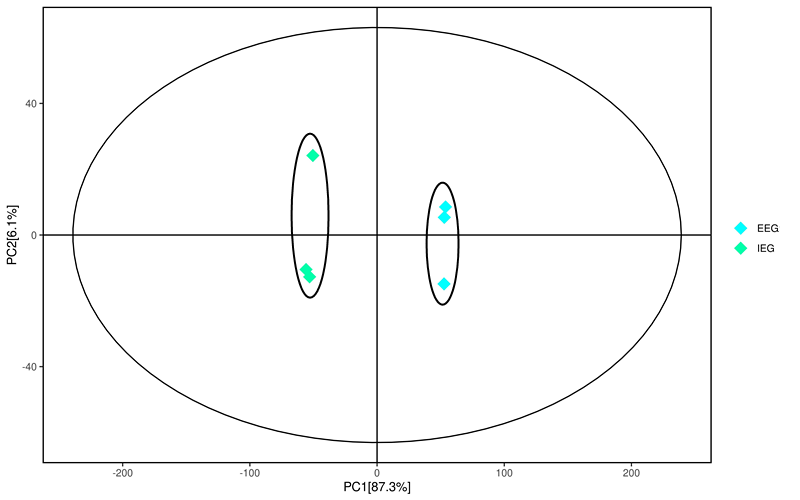


**Fig. S2.** PCA analysis for groups EEG and IEG. IEG, EEG represent intracellular and extracellular metabolites, respectively.


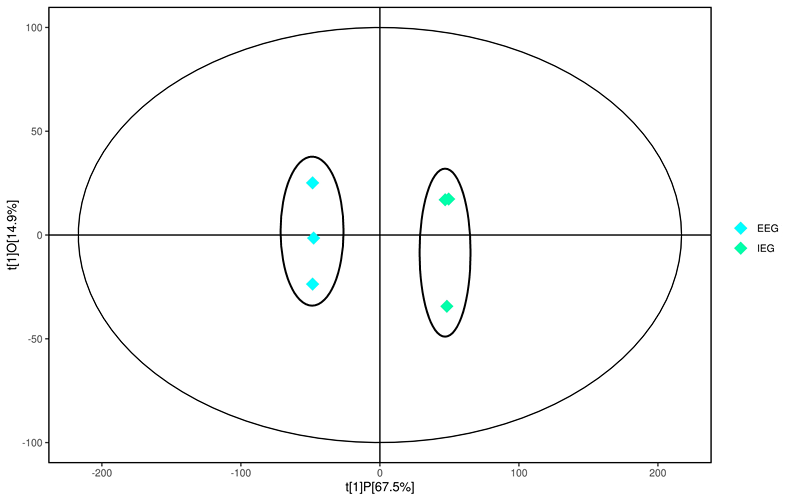


**Fig. S3.** OPLS-DA analysis for group EEG and IEG. IEG, EEG represent intracellular and extracellular metabolites, respectively.


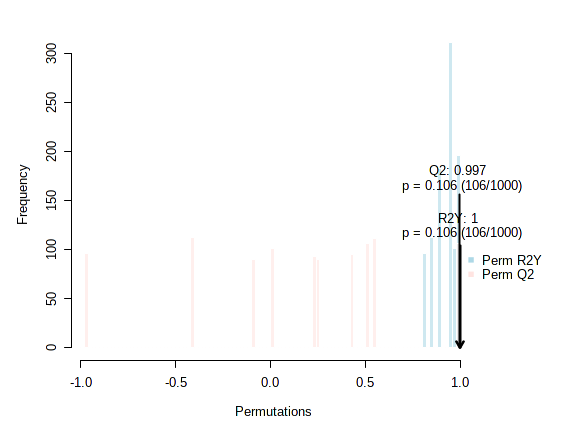


**Fig. S4.** Permutation test for group EEG and IEG. IEG, EEG represent intracellular and extracellular metabolites, respectively.


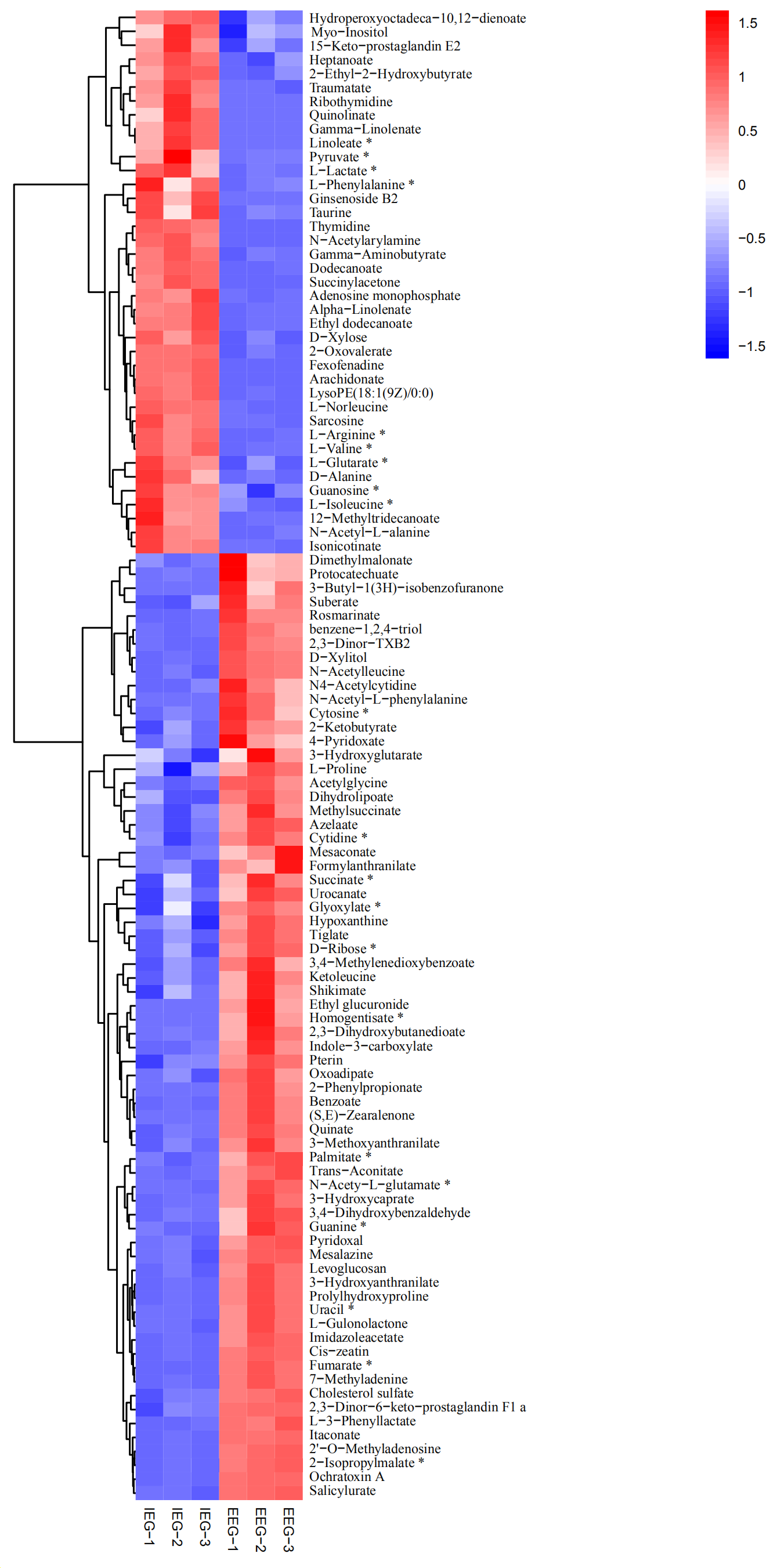


**Fig. S5.** Heatmap of hierarchical clustering analysis for group EEG and IEG. IEG, EEG represent intracellular and extracellular metabolites, respectively; * represents the metabolite can be annotated in KEGG database.
